# Neoadjuvant compared to adjuvant chemotherapy combined with trastuzumab in patients with HER2-positive breast cancer: a register-based cohort study

**DOI:** 10.1016/j.esmorw.2024.100093

**Published:** 2024-11-19

**Authors:** S. Hosseini-Mellner, Å. Wickberg, E. Olsson, A. Karakatsanis, A. Valachis

**Affiliations:** 1 School of Medicine, Faculty of Medicine and Health, Örebro University, Örebro, Sweden; 2 Department of Surgery, Stockholm South General Hospital, Stockholm, Sweden; 3 Department of Surgery, Faculty of Medicine and Health, Örebro University Hospital, Örebro University, Örebro, Sweden; 4 Department of Immunology, Genetics, and Pathology, Uppsala University, Uppsala, Sweden; 5 Department of Surgical Sciences, Uppsala University Hospital, Uppsala University, Uppsala, Sweden; 6 Section for Breast Surgery, Department of Surgery, Uppsala University Hospital, Uppsala, Sweden; 7 Department of Oncology, Faculty of Medicine and Health, Örebro University Hospital, Örebro University, Örebro, Sweden

**Keywords:** breast cancer, HER2-positive, neoadjuvant, adjuvant, real-world evidence

## Abstract

**Background:**

The aim of the study was to compare trastuzumab-based neoadjuvant therapy (NAT) with adjuvant therapy (AT) in a register-based cohort of Swedish patients with primary operable human epidermal growth factor receptor 2 (HER2)-positive breast cancer.

**Patients and methods:**

The Swedish nationwide research database BCBaSe 3.0 was used to identify eligible patients with primary operable HER2-positive breast cancer treated with either NAT or AT between 2008 and 2019. To mitigate confounding by indication bias, propensity score matching (PSM) and inverse probability of treatment weighting (IPTW) were applied.

**Results:**

In total, 7258 patients with primary operable HER2-positive breast cancer were identified; 1789 (24.6%) received NAT and 5469 (75.4%), AT. After 1 : 1 PSM, 1258 patients in each therapeutic strategy were available for comparisons. No statistically significant differences between NAT and AT were observed [hazard ratio (HR) for distant disease-free survival 0.97, 95% confidence (CI) 0.72-1.30; HR for breast cancer-specific survival (BCSS) 0.69, 95% CI 0.45-1.07; HR for overall (OS) 0.72, 95% CI 0.50-1.05]. In subgroup analysis, NAT resulted in better BCSS (HR 0.44, 95% CI 0.22-0.89) and OS (HR 0.49, 95% CI 0.29-0.90) in patients with clinical node positivity (cN+) at diagnosis.

**Conclusion:**

The study shows an equivalence of NAT and AT in terms of prognosis for patients with operable HER2-positive disease whereas a potential benefit of NAT in patients with cN+ is implied. Considering the emerging treatment strategies in the neoadjuvant setting for HER2-positive breast cancer that are not reflected in the study cohort, NAT should be considered as the strategy with a higher possibility of improving long-term prognosis for patients with HER2-positive disease.

## Highlights

• We found similar prognosis in HER2-positive breast cancer treated with NAT versus AT.
• A potential benefit of neoadjuvant therapy in clinically positive lymph node status can be implied.
• Neoadjuvant therapy seems to offer a higher possibility of improving prognosis in HER2-positive disease.

## Introduction

A clear trend towards increased use of neoadjuvant therapy (NAT) compared to adjuvant therapy (AT) in patients with operable human epidermal growth factor receptor 2 (HER2)-positive breast cancer has been observed in the past decade.^1^^,^^2^ This trend reflects the similar survival benefit with either treatment strategy^3^ and the advantages associated with neoadjuvant therapy in terms of surgical de-escalation^4^ and a more individualized treatment strategy in the adjuvant setting based on the magnitude of response to NAT.^5^

Despite the theoretical advantages of NAT in patients with HER2-positive breast cancer, there is no compelling direct evidence that supports a survival benefit compared to AT. Some indirect evidence implies that early initiation of systemic anti-HER2 treatment (as in the neoadjuvant setting) in patients with operable HER2-positive breast cancer might be beneficial.^6^^,^^7^ Few studies have compared NAT with AT, showing no survival benefit with either treatment strategy, but the relatively low number of patients and the single-center nature of data sources influence the validity and generalizability of study results.^8^, ^9^, ^10^

The purpose of this study was to investigate the survival outcomes between patients with operable HER2-positive breast cancer treated with trastuzumab-based NAT and AT using a nationwide, population-based cohort from Sweden.

## Patients and methods

The study is reported in accordance with the European Society for Medical Oncology-Guidance for Reporting Oncology real-world evidence reporting guidance for oncology real-world evidence studies.^11^

### Study design

We used the research database BCBaSe 3.0 as data source and carried out a retrospective, registry- and population-based, cohort study.

### Data source

The research database BCBaSe 3.0 is based on the Swedish National Quality Register for Breast Cancer (Nationell Kvalitetsregister för bröstcancer; NKBC), where all patients with newly diagnosed breast cancer in Sweden are registered since 2008. BCBaSe 3.0 consists of the linkage between NKBC and other relevant national registries through the unique Swedish national identification number that is assigned to every individual listed in the Swedish Population Register.

For the purpose of the present study, we utilized relevant data from NKBC, the Cause of Death Register,^12^ and the Longitudinal Integration Database for Health Insurance and Labour Market Studies (LISA) including information on patients’ socioeconomic status.^13^

### Study cohort

Patients with primary operable HER2-positive breast cancer diagnosed from the inception of NKBC from 2008 until December 2019 and treated with neoadjuvant or adjuvant trastuzumab-based chemotherapy were identified and included in the study cohort.

We excluded patients with cT4 and/or cN3 disease or inflammatory breast cancer, patients with *de novo* metastatic disease (within 3 months from breast cancer diagnosis), as well as patients who did not receive chemotherapy and/or trastuzumab at any time during curative treatment.

Considering the timelines for reimbursement of pertuzumab as neoadjuvant (March 2016; restricted only to locally advanced HER2-positive breast cancer) and T-DM1 as post-neoadjuvant treatment (March 2020), only a limited number of patients in the study cohort are expected to be treated with these treatment strategies.

### Definitions and outcomes

HER2 positivity was defined as either the presence of 3+ at immunohistochemistry analysis or 2+ with HER2 amplification on *in situ* hybridization. For estrogen receptor (ER) positivity, a cut-off of 10% in immunohistochemistry analysis was used considering the similar prognosis between ER-low positive (ER 1%-9%) and ER-negative (ER < 1%) disease according to several studies.^14^

The following time-to-event outcomes were used as relevant survival outcomes: distant disease-free survival (DDFS), breast cancer-specific survival (BCSS), and overall survival (OS). All three outcomes were defined according to STEEP 2.0.^15^ Specifically, DDFS was defined as the time from breast cancer diagnosis until the occurrence of distant metastasis or death due to any cause; BCSS was defined as the time from breast cancer diagnosis until death due to breast cancer; OS was defined as the time from breast cancer diagnosis until death due to any cause.

### Statistical analyses

Continuous variables were presented as median and range whereas categorical variables as numbers and percentages.

The chi-square test or Mann–Whitney test was used to compare baseline characteristics between the two treatment groups (NAT versus AT).

Propensity score matching (PSM) was used to mitigate the risk of confounding by indication, namely NAT might have been selected for breast cancer patients with more advanced and aggressive tumors. Propensity scores were estimated by using logistic regression model with variables that were considered to potentially impact the treatment decision including age at diagnosis, gender, cT stage, cN stage, tumor grade, geographical region, and income.

We used a 1 : 1 ‘nearest neighbor’ matching method without replacement to compare the difference between neoadjuvant chemotherapy and adjuvant treatment. The comparability of baseline characteristics between the two treatment groups after matching was assessed using the chi-square test or the Mann–Whitney test as appropriate.

Multivariate Cox proportional hazards regression models were carried out to assess independent factors associated with survival outcomes in the PSM cohort including predefined variables (age, sex, cT, cN, region, income, ER status, surgery type in breast and axilla, post-operative radiotherapy) as covariates and treatment setting (neoadjuvant versus adjuvant) as the variable of interest. Complete case analysis was applied to all Cox regression models.

As sensitivity analyses, we carried out multivariate Cox proportional hazards models for each survival outcome using the pre-PSM cohort but including the inverse probability of treatment weighting (IPTW) as a covariate in the models. An additional sensitivity analysis for DDFS was carried out including only patients treated in health care region of Stockholm–Gotland region, where the completeness of recurrence data is considerably higher compared to the completeness in NKBC overall.

Subgroup analyses, reflecting patient subgroups of special interest (ER-positive versus ER-negative disease; cN+ versus cN0 disease), were carried out using data from the PSM cohort. Within each subgroup analysis, Cox proportional hazards regression models for each survival outcome were carried out.

## Results

### Patient selection process and baseline characteristics

**Figure 1:**
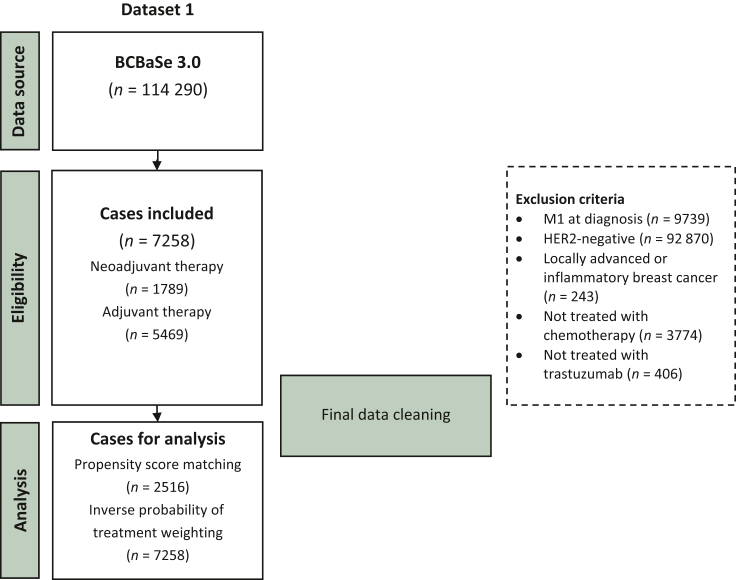
Flowchart diagram of the patient selection process.

In total, 7258 patients with primary operable HER2-positive breast cancer treated with either NAT or AT including chemotherapy and trastuzumab-based anti-HER2 treatment were identified from the BCBaSe 3.0 after applying the inclusion and exclusion criteria (Figure 1). Among the included patients, 1789 (24.6%) received NAT and 5469 (75.4%), AT. Table 1 describes patient-, tumor-, and treatment-related characteristics of the study cohort based on treatment setting. Patients treated with NAT were in general younger with more advanced disease at diagnosis, and they underwent more extensive surgical procedures compared to patients treated with AT. Some geographical disparities were also observed with higher NAT utilization in the Stockholm–Gotland region.

**Table 1:** Baseline characteristics between treatment groups before propensity score matching

Characteristics	Neoadjuvant chemotherapy plus trastuzumab *N* = 1789; *n* (%)	Adjuvant chemotherapy plus trastuzumab *N* = 5469; *n* (%)	*P* value
Age, median (Q1-Q3)	52 (43-62)	59 (49-67)	<0.001
Sex			0.028
Male	3 (0.2)	31 (0.6)	
Female	1786 (99.8)	5438 (99.4)	
Household income			<0.001
Quartile 1 (lowest)	661 (37.4)	1632 (30.0)	
Quartile 2	458 (25.9)	1526 (28.1)	
Quartile 3	358 (20.2)	1345 (24.8)	
Quartile 4 (highest)	291 (16.5)	928 (17.1)	
Region of residency			<0.001
North	129 (7.3)	501 (9.2)	
Stockholm—Gotland	642 (36.1)	1127 (20.7)	
Uppsala-Örebro	214 (12.0)	1037 (19.1)	
South	330 (18.6)	892 (16.4)	
Southeast	183 (10.3)	643 (11.8)	
West	278 (15.7)	1243 (22.8)	
cT			<0.001
T1	308 (17.2)	3387 (61.9)	
T2 or T3	1481 (82.8)	2082 (38.1)	
cN			<0.001
N0	811 (45.3)	4679 (85.6)	
N1 or N2	967 (54.1)	779 (14.2)	
Anatomic stage			<0.001
I	132 (7.3)	2544 (46.5)	
II	1331 (74.5)	2262 (41.4)	
III	326 (18.2)	663 (12.1)	
Histology			0.029
Ductal	438 (89.2)	5012 (92.5)	
Lobular	32 (6.5)	234 (4.3)	
Other	21 (4.3)	171 (3.2)	
Missing	1298	52	
ER status			<0.001
Positive	1029 (57.5)	3652 (66.8)	
Negative	760 (42.5)	1817 (33.2)	
Tumor grade			<0.001
I	56 (10.5)	128 (2.4)	
II	274 (51.4)	1762 (32.8)	
III	203 (38.1)	3480 (64.8)	
Missing	1256	99	
Ki-67, median (Q1-Q3)	22 (7-41.5)	37 (25-50)	<0.001
Missing	1296	1451	
Surgery of primary tumor			<0.001
Breast conserving surgery	663 (37.1)	3075 (56.2)	
Mastectomy	1083 (60.5)	2393 (43.8)	
Axillary surgery			<0.001
SLND	464 (27.3)	3585 (66.9)	
ALND	1039 (61.0)	875 (16.3)	
SLND ≥ ALND	199 (11.7)	901 (16.8)	
Radiotherapy			<0.001
No	285 (15.9)	1318 (24.1)	
Yes, breast or chest wall	392 (21.9)	2618 (47.9)	
Yes, breast and lymph nodes	1112 (62.2)	1533 (28.0)	
Endocrine therapy			<0.001
No	760 (42.5)	1817 (33.2)	
Yes	1029 (57.5)	3652 (66.8)	

ALND, axillary lymph node dissection; cN, clinical node status; cT, clinical tumor status; ER, estrogen receptor; SLND, sentinel lymph node dissection.

### Baseline characteristics after propensity score matching

After 1 : 1 PSM, 1258 patients in each treatment strategy were available for comparisons. The differences in baseline characteristics were diminished but some differences in terms of extent of axillary surgery and radiotherapy between the two groups remained (Table 2).

**Table 2:** Baseline characteristics between treatment groups after propensity score matching

Characteristics	Neoadjuvant chemotherapy plus trastuzumab *N* = 1258; *n* (%)	Adjuvant chemotherapy plus trastuzumab *N* = 1258; *n* (%)	*P* value
Age, median (Q1-Q3)	55 (45-64)	55 (46-64)	1.000
Sex			1.000
Male	3 (0.2)	2 (0.2)	
Female	1255 (99.8)	1256 (99.8)	
Household income			0.846
Quartile 1 (lowest)	453 (36.0)	434 (34.5)	
Quartile 2	332 (26.4)	339 (26.9)	
Quartile 3	265 (21.1)	265 (21.1)	
Quartile 4 (highest)	208 (16.5)	220 (17.5)	
Region of residency			0.996
North	93 (7.4)	92 (7.3)	
Stockholm—Gotland	393 (31.2)	398 (31.6)	
Uppsala-Örebro	183 (14.5)	186 (14.8)	
South	228 (18.1)	230 (18.3)	
Southeast	142 (11.3)	133 (10.6)	
West	219 (17.4)	219 (17.4)	
cT			0.067
T1	294 (23.4)	256 (20.3)	
T2 or T3	964 (76.6)	1002 (79.7)	
cN			0.513
N0	749 (59.5)	777 (61.8)	
N1 or N2	502 (39.9)	475 (37.8)	
Anatomic stage			0.789
I	131 (10.4)	134 (10.7)	
II	901 (71.6)	911 (72.4)	
III	226 (18.0)	213 (16.9)	
ER status			0.616
Positive	776 (61.9)	765 (61.0)	
Negative	477 (38.1)	490 (39.0)	
Surgery of primary tumor			0.744
Breast conserving surgery	486 (39.8)	508 (40.4)	
Mastectomy	736 (60.2)	749 (59.6)	
Axillary surgery			<0.001
SLND	422 (35.6)	576 (46.7)	
ALND	613 (51.7)	424 (34.4)	
SLND ≥ ALND	150 (12.7)	233 (18.9)	
Radiotherapy			<0.001
No	249 (19.8)	275 (21.9)	
es, breast or chest wall	335 (26.6)	414 (32.9)	
Yes, breast and lymph nodes	674 (53.6)	569 (45.2)	
Endocrine therapy			0.074
No	528 (42.0)	484 (38.5)	
Yes	730 (58.0)	774 (61.5)	

ALND, axillary lymph node dissection; cN, clinical node status; cT, clinical tumor status; ER, estrogen receptor; SLND, sentinel lymph node dissection.

### Impact of neoadjuvant compared to adjuvant therapy on survival outcomes

After a median follow-up of 63 months, no statistically significant differences between NAT and AT were observed in either outcome [hazard ratio (HR) for DDFS 0.94, 95% confidence interval (CI) 0.70-1.27; HR for BCSS 0.69, 95% CI 0.45-1.07; HR for OS 0.72, 95% CI 0.50-1.05] (Table 3). The results remained unchanged in sensitivity analysis when the overall study cohort was analyzed and IPTW adjustments were applied (Table 3). Similarly, the DDFS-specific sensitivity analysis with the restriction of study cohort only to patients treated in the Stockholm–Gotland region showed similar results with the main DDFS analysis (Table 3).

**Table 3:** Multivariable Cox proportional hazards regression analyses for survival outcomes between treatment groups (neoadjuvant versus adjuvant) after propensity score matching including sensitivity analyses

Outcome	Adjusted hazard ratio^a^	95% confidence interval
Adjuvant (reference) versus neoadjuvant chemotherapy		
Distant disease-free survival (PSM cohort)	0.94	0.70-1.27
Breast cancer-specific survival (PSM cohort)	0.69	0.45-1.07
Overall survival (PSM cohort)	0.72	0.50-1.05
Distant disease-free survival (PSM cohort; sensitivity analysis including only patients from Stockholm—Gotland)	0.88	0.54-1.51
Distant disease-free survival (sensitivity analysis^b^)	0.92	0.70-1.20
Breast cancer-specific survival (sensitivity analysis^b^)	0.81	0.57-1.16
Overall survival (sensitivity analysis^b^)	0.77	0.57-1.05

cN, clinical node status; cT, clinical tumor status; IPTW, inverse probability of treatment weighting; PSM, propensity score matching.

a Adjusted for age, sex, cT, cN, region, income, estrogen receptor status, surgery type in breast and axilla, post-operative radiotherapy.

b Sensitivity analysis using the pre-PSM cohort and including IPTW into the adjusted models. Same variables as above were used as covariates.

### Subgroup analyses

In subgroup analyses, ER status did not impact the survival outcomes in the two treatment settings (Table 4). When analyses were restricted to patients with cN+ disease, NAT resulted in longer BCSS (HR 0.44, 95% CI 0.22-0.89) and OS (HR 0.49, 95% CI 0.29-0.90; Table 4) compared with AT. The latter trend was not evident in patients with cN0 disease (Table 4).

**Table 4:** Subgroup analyses for survival outcomes between treatment groups (neoadjuvant versus adjuvant) after propensity score matching

Outcome	Adjusted hazard ratio	95% confidence interval
Adjuvant (reference) versus neoadjuvant chemotherapy		
ER-positive disease (*n* = 1541)^a^		
Distant disease-free survival	0.82	0.55-1.21
Breast cancer-specific survival	0.79	0.42-1.50
Overall survival	0.57	0.33-1.00
ER-negative disease (*n* = 967)^a^		
Distant disease-free survival	0.89	0.60-1.34
Breast cancer-specific survival	0.64	0.36-1.16
Overall survival	0.88	0.54-1.46
cN-negative disease (*n* = 1526)^b^		
Distant disease-free survival	1.03	0.70-1.49
Breast cancer-specific survival	0.84	0.48-1.47
Overall survival	0.80	0.50-1.29
cN-positive disease (*n* = 977)^b^		
Distant disease-free survival	0.82	0.52-1.31
Breast cancer-specific survival	0.44	0.22-0.89
Overall survival	0.49	0.27-0.90

cN, clinical node status; cT, clinical tumor status; ER, estrogen receptor.

a Adjusted for age, sex, cT, cN, region, income, surgery type in breast and axilla, post-operative radiotherapy.

b Adjusted for age, sex, cT, region, income, ER status, surgery type in breast and axilla, post-operative radiotherapy.

## Discussion

The study results reflect the real-world prognosis of patients with operable HER2-positive breast cancer treated with trastuzumab-based NAT or AT in the pre-post-neoadjuvant therapy era and suggest an equivalence of the two treatment strategies in terms of prognosis. Although ER status does not seem to influence the potential benefit of either treatment strategy, NAT compared with AT seems to be beneficial in terms of survival in patients with clinically positive lymph node status.

Our findings are in line with the results from previous real-world evidence (RWE) studies where similar survival outcomes between patients treated with trastuzumab-based NAT and AT have been reported.^8^, ^9^, ^10^ Considering the risk for indication bias between the two treatment groups, all previous studies applied PS methods with either PSM^8^ or IPTW^9^^,^^10^ to limit the risk of bias. However, we chose to apply both methods (PSM as main analysis; IPTW as sensitivity analysis) recognizing the high risk for the presence of unobserved confounding where multiple PS approaches are recommended to strengthen the validity of study results.^16^ Apart from that, our study comprises a considerably larger, nationwide, and population-based cohort with longer median follow-up (>5 years) compared to previous single-center RWE studies with l<5 years of follow-up,^8^, ^9^, ^10^ thus increasing the external validity of the results.

Two well-documented and clinically relevant markers were chosen for stratified analyses, namely ER positivity and lymph node status as ER positivity is prognostic within HER2-positive disease^17^ and positive lymph node status at diagnosis remains one of the most impactful negative prognostic factors in breast cancer even in the modern era^18^. Our stratified analysis revealed a survival benefit of NAT compared to AT in patients with positive lymph node status, whereas such a trend was not evident in lymph node-negative disease. Our results are in line with previous RWE where survival benefit with NAT in patients with positive lymph nodes was observed.^9^^,^^10^ As lymph node positivity suggests more advanced disease, earlier initiation of systemic anti-HER2-based therapy as NAT might be a possible explanation for this survival trend. In fact, delay in administration of anti-HER2-based therapy is found to be associated with worse prognosis in patients with HER2-positive breast cancer.^6^^,^^7^ From a biological perspective, a potential survival benefit of NAT in biologically and anatomically more aggressive disease might be associated with trastuzumab’s immune-mediated mode of action through antibody-dependent cellular cytotoxicity that could theoretically be more effective in the presence of tumor.^19^

The interpretation of study results should acknowledge the treatment strategies applied in the study cohort that differ from current clinical practice.^20^ Specifically, dual HER2 blockade with trastuzumab and pertuzumab was approved in Sweden in 2016 with restriction to be used only in patients with locally advanced breast cancer so most of the patients treated with NAT in the study cohort only received trastuzumab combined with chemotherapy. In addition, post-neoadjuvant trastuzumab emtansine that has been shown to improve survival in patients with HER2-positive breast cancer and residual disease after NAT^21^ was not available for this indication before 2020. It is, therefore, reasonable to argue that the implementation of these treatment strategies in the neoadjuvant setting would probably have implications for the prognosis of patients with HER2-positive breast cancer treated with NAT that would be expected to be better compared to AT in our study cohort.

An interesting, though exploratory, observation derived from our study results that deserves attention is the impact of NAT on surgical de-escalation strategies, which is feasible yet not fully utilized.^22^ As expected, more radical surgical procedures such as mastectomy and axillary lymph node dissection (ALND) were carried out in patients treated with NAT, mainly reflecting that patients with anatomically more advanced disease were selected to be treated with NAT. When PSM was applied, thus diminishing the differences in anatomic staging between the two treatment strategies, the frequency of mastectomies was comparable between NAT and AT, although one could expect less mastectomies in the NAT group. Besides, substantially more ALND procedures were carried out in patients treated with NAT both before and after PSM. It seems, therefore, that the surgical benefits of NAT regarding de-escalation of breast and axillary surgery^23^, ^24^, ^25^ have not been implemented into clinical practice for this patient cohort. Despite the explorative nature of this observation, our study results seem to imply an unmet need for implementing de-escalation strategies on breast and axillary surgery in patients with HER2-positive breast cancer treated with NAT.

The study results should be interpreted with caution considering some limitations related to the nature of study design and data sources but also some strengths. Firstly, the treatment allocation to NAT or AT was based on clinical decision that was assessed retrospectively, thus introducing a high risk for indication bias. Although we tried to limit the risk of bias through applying two different PS methods to strengthen the validity of our results, there is still a considerable risk for residual bias. Furthermore, there have been some concerns on the reliability of data on distant recurrence in BCBaSe 3.0. However, a sensitivity analysis restricted only to patients diagnosed at the Stockholm–Gotland health care region, where data on distant recurrence have been validated, showed similar results to main analyses, thus strengthening the reliability of DDFS as an outcome in the whole study cohort. An additional limitation is that the neoadjuvant therapeutic approach in the study cohort did not include some emerging treatment strategies that are currently considered standard of care such as dual HER2 blockade as a part of the neoadjuvant therapeutic scheme and trastuzumab emtansine as post-neoadjuvant treatment in case of residual disease. Despite these caveats, the relatively large number of eligible patients that enables stratified analyses based on clinically relevant parameters and the population-based nature of data source offer the opportunity for observations of clinical relevance that reflect NAT patterns and effectiveness in the real-world setting with results that can be generalized to a broader context.

## Conclusion

In summary, the study results suggest similar survival outcomes between NAT and AT in patients with operable HER2-positive disease irrespective of ER status whereas a potential benefit of NAT compared to AT in patients with clinically positive lymph node status is implied. Considering the emerging treatment strategies in the neoadjuvant setting for HER2-positive breast cancer that are not reflected in the study cohort, NAT should be considered as the strategy with a higher possibility of improving long-term prognosis for patients with HER2-positive disease.

## References

[1] Ortmann O., Blohmer J.U., Sibert N.T. (2023). Current clinical practice and outcome of neoadjuvant chemotherapy for early breast cancer: analysis of individual data from 94,638 patients treated in 55 breast cancer centers. J Cancer Res Clin Oncol.

[2] Prakash I., Neely N.B., Thomas S.M. (2022). Utilization of neoadjuvant chemotherapy in high-risk, node-negative early breast cancer. Cancer Med.

[3] Early Breast Cancer Trialists’ Collaborative Group (EBCTCG) (2018). Long-term outcomes for neoadjuvant versus adjuvant chemotherapy in early breast cancer: meta-analysis of individual patient data from ten randomised trials. Lancet Oncol.

[4] Shubeck S.P., Morrow M., Dossett L.A. (2022). De-escalation in breast cancer surgery. NPJ Breast Cancer.

[5] von Minckwitz G., Huang C.S., Mano M.S. (2019). Trastuzumab emtansine for residual invasive HER2-positive breast cancer. N Engl J Med.

[6] Gullo G., Walsh N., Fennelly D. (2018). Impact of timing of trastuzumab initiation on long-term outcome of patients with early-stage HER2-positive breast cancer: the “one thousand HER2 patients” project. Br J Cancer.

[7] Gallagher C.M., More K., Kamath T. (2016). Delay in initiation of adjuvant trastuzumab therapy leads to decreased overall survival and relapse-free survival in patients with HER2-positive non-metastatic breast cancer. Breast Cancer Res Treat.

[8] Zheng S., Li L., Chen M. (2022). Benefits of neoadjuvant therapy compared with adjuvant chemotherapy for the survival of patients with HER2-positive breast cancer: a retrospective cohort study at FUSCC. Breast.

[9] Pomponio M.K., Burkbauer L., Goldbach M. (2020). Refining the indications for neoadjuvant chemotherapy for patients with HER2+ breast cancer: a single institution experience. J Surg Oncol.

[10] Laas E., Bresset A., Féron J.G. (2021). *HER2*-positive breast cancer patients with pre-treatment axillary involvement or postmenopausal status benefit from neoadjuvant rather than adjuvant chemotherapy plus trastuzumab regimens. Cancers (Basel).

[11] Castelo-Branco L., Pellat A., Martins-Branco D. (2023). ESMO Guidance for Reporting Oncology real-world evidence (GROW). Ann Oncol.

[12] Brooke H.L., Talbäck M., Hörnblad J. (2017). The Swedish cause of death register. Eur J Epidemiol.

[13] Ludvigsson J.F., Svedberg P., Olén O., Bruze G., Neovius M. (2019). The longitudinal integrated database for health insurance and labour market studies (LISA) and its use in medical research. Eur J Epidemiol.

[14] Paakkola N.M., Karakatsanis A., Mauri D., Foukakis T., Valachis A. (2021). The prognostic and predictive impact of low estrogen receptor expression in early breast cancer: a systematic review and meta-analysis. ESMO Open.

[15] Tolaney S.M., Garrett-Mayer E., White J. (2021). Updated standardized definitions for efficacy end points (STEEP) in adjuvant breast cancer clinical trials: STEEP version 2.0. J Clin Oncol.

[16] Ciminata G., Geue C., Wu O., Deidda M., Kreif N., Langhorne P. (2022). Propensity score methods for comparative-effectiveness analysis: a case study of direct oral anticoagulants in the atrial fibrillation population. PLoS One.

[17] Han Y., Wu Y., Xu H., Wang J., Xu B. (2022). The impact of hormone receptor on the clinical outcomes of HER2-positive breast cancer: a population-based study. Int J Clin Oncol.

[18] Johansson A.L.V., Trewin C.B., Fredriksson I., Reinertsen K.V., Russnes H., Ursin G. (2021). In modern times, how important are breast cancer stage, grade and receptor subtype for survival: a population-based cohort study. Breast Cancer Res.

[19] Gennari R., Menard S., Fagnoni F. (2004). Pilot study of the mechanism of action of preoperative trastuzumab in patients with primary operable breast tumors overexpressing HER2. Clin Cancer Res.

[20] Loibl S., André F., Bachelot T. (2024). ESMO Guidelines Committee. Early breast cancer: ESMO Clinical Practice Guideline for diagnosis, treatment and follow-up. Ann Oncol.

[21] von Minckwitz G., Huang C.S., Mano M.S., KATHERINE Investigators (2019). Trastuzumab emtansine for residual invasive HER2-positive breast cancer. N Engl J Med.

[22] Karakatsanis A., Tasoulis M.K., Wärnberg F., Nilsson G., MacNeill F. (2018). Meta-analysis of neoadjuvant therapy and its impact in facilitating breast conservation in operable breast cancer. Br J Surg.

[23] Petruolo O., Sevilimedu V., Montagna G., Le T., Morrow M., Barrio A.V. (2021). How often does modern neoadjuvant chemotherapy downstage patients to breast-conserving surgery?. Ann Surg Oncol.

[24] Kuemmel S., Heil J., Rueland A. (2022). A prospective, multicenter registry study to evaluate the clinical feasibility of targeted axillary dissection (TAD) in node-positive breast cancer patients. Ann Surg.

[25] Kuemmel S., Heil J., Bruzas S. (2023). Safety of targeted axillary dissection after neoadjuvant therapy in patients with node-positive breast cancer. JAMA Surg.

